# Strain control of oxygen kinetics in the Ruddlesden-Popper oxide La_1.85_Sr_0.15_CuO_4_

**DOI:** 10.1038/s41467-017-02568-z

**Published:** 2018-01-08

**Authors:** Tricia L. Meyer, Ryan Jacobs, Dongkyu Lee, Lu Jiang, John W. Freeland, Changhee Sohn, Takeshi Egami, Dane Morgan, Ho Nyung Lee

**Affiliations:** 1 Materials Science and Technology Division, Oak Ridge National Laboratory, Oak Ridge, TN 37831 USA; 2 Department of Materials Science and Engineering, University of Wisconsin-Madison, Madison, 53706 WI USA; 3 Department of Physics and Astronomy, University of Tennessee, Knoxville, TN 37996 USA; 4 Advanced Photon Source, Argonne National Laboratory, Argonne, 60439 IL USA; 5 Department of Materials Science and Engineering, University of Tennessee, Knoxville, TN 37996 USA

## Abstract

Oxygen defect control has long been considered an important route to functionalizing complex oxide films. However, the nature of oxygen defects in thin films is often not investigated beyond basic redox chemistry. One of the model examples for oxygen-defect studies is the layered Ruddlesden–Popper phase La_2**−***x*_Sr_*x*_CuO_4**−***δ*_ (LSCO), in which the superconducting transition temperature is highly sensitive to epitaxial strain. However, previous observations of strain-superconductivity coupling in LSCO thin films were mainly understood in terms of elastic contributions to mechanical buckling, with minimal consideration of kinetic or thermodynamic factors. Here, we report that the oxygen nonstoichiometry commonly reported for strained cuprates is mediated by the strain-modified surface exchange kinetics, rather than reduced thermodynamic oxygen formation energies. Remarkably, tensile-strained LSCO shows nearly an order of magnitude faster oxygen exchange rate than a compressively strained film, providing a strategy for developing high-performance energy materials.

## Abstract (web summary)

The desirable functional properties of complex oxide materials are often influenced by the presence of oxygen defects and epitaxial strain. Meyer et al. demonstrate the role of oxygen defect kinetics in the strain control of the superconducting transition temperature of LSCO.

## Introduction

As witnessed over the last few decades, the fundamental properties of complex oxides are closely related to their oxygen stoichiometry. Examples include the critical temperature (*T*_c_) in superconductors, oxygen-defect-mediated conduction in memristors, two dimensional electron gas at interfaces, and hole doping in correlated oxides for colossal magnetoresistance^1–4^. This stoichiometry dependence is attributed to the fact that the creation or annihilation of oxygen vacancies can change the charge carrier concentration and hence modify materials’ electronic, magnetic, or ionic properties^5^. Excellent examples of this property tuning can be found in studies of the superconducting phases in bulk La_2−*x*_Sr_*x*_CuO_4−*δ*_ (LSCO), in which the superconductivity and the associated *T*_c_ are highly sensitive to the number of hole carriers chemically doped by changing the strontium concentration, *x*, or oxygen nonstoichiometry, *δ*^1, 6, 7^.

Similar to carrier density tuning, strain engineering plays a leading role in the advancement of electronic devices by improving physical properties^8–11^. An important discovery resulting from strain engineering was the existence of strong coupling between the strain state and the *T*_c_ for superconductivity in LSCO thin films^12–14^. For LSCO, enhancement (or suppression) of the *T*_c_ was realized by inducing compressive (or tensile) strain. However, the previous understanding of the relationship between strain and *T*_c_ must be re-examined, because recent studies have found that strain, especially tensile strain, significantly lowers the thermodynamically driven oxygen vacancy formation energy^15–17^. Since the latter experimental observations are closely linked with the post-annealing conditions in oxygen-rich or oxygen-deficient atmospheres, consideration of both thermodynamics and kinetics is pivotal for an accurate understanding of the origin of strain-driven phenomena—not only in these superconducting oxides but also potentially in the many functional complex oxides in which oxygen stoichiometry directly influences physical properties.

In this work, we used La_1.85_Sr_0.15_CuO_4_ epitaxial films to investigate the kinetic and thermodynamic contributions to strain-driven phenomena. Since the superconducting *T*_c_ is highly sensitive to oxygen stoichiometry, both tensile-strained and compressive-strained films were studied to systematically illustrate the strain coupling with oxygen nonstoichiometry and superconductivity. Density functional theory (DFT) calculations were also performed to calculate the oxygen-defect energetics, and an analysis of experimentally derived surface exchange kinetics was used to account for strain-induced oxygen loss in tensile-strained films.

## Results

### Strain-dependent transport properties

To investigate the correlations between strain and oxygen nonstoichiometry, we deposited epitaxial LSCO (*a = *3.777 Å, *c* = 13.226 Å)^1^ films on various single-crystalline substrates via pulsed laser epitaxy to modulate the epitaxial strain. The substrates included LaSrAlO_4_ (LSAO, *a* = 3.756 Å), LaAlO_3_ (LAO, *a* = 3.788 Å), (LaAlO_3_)_0.3_(SrAl_0.5_Ta_0.5_O_3_)_0.7_ (LSAT, *a* = 3.868 Å), and SrTiO_3_ (STO, *a = *3.905 Å), which induced biaxial strains of −0.61, 0.29, 2.35, and 3.28%, respectively. To isolate the influence of strain from changes in oxygen content and discriminate between the kinetic and thermodynamic contributions, thin films grown on LAO (tensile strain) and LSAO (compressive strain) were annealed in both reducing and oxidizing atmospheres. Confirmations of the strain state and high-quality growth of these films were completed using X-ray diffraction (XRD), shown in Supplementary Fig. 1.

**Fig. 1:**
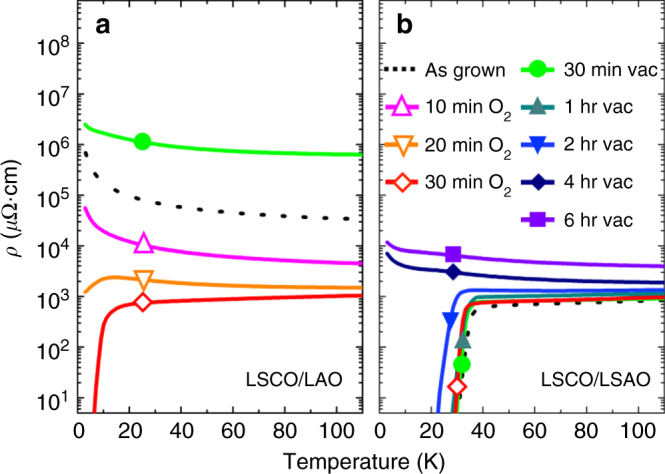
Strain and redox control of superconductivity. Temperature-dependent resistivity of as-grown, vacuum-annealed (PO_2_ = 10^−6^ Torr), and oxygen-annealed (PO_2_ = 100 Torr) LSCO films (20 nm in thickness) on LAO with + 0.29% tensile strain (**a**) and on LSAO with −0.61% compressive strain (**b**). Symbols on each curve represent the annealing conditions. Note the annealing temperature was fixed at 500 °C. **a** Tensile-strained films show a drastic reduction in resistivity after annealing in oxygen for a short time at 500 °C, indicating the fast removal of oxygen vacancies under tensile strain. Vacuum annealing further increases the resistivity, which indicates the creation of more oxygen vacancies. **b** LSCO films under compression exhibit robust superconductivity (*T*_c_ ~ 31 K) for as-grown and oxygen-annealed samples. Vacuum annealing for up to 2 h did not significantly alter the oxygen stoichiometry. However, longer annealing (>2 h) eventually removed the superconductivity as a result of additional oxygen vacancy creation. This long annealing time result indicates that oxygen kinetics play a central role in oxygen loss, with compressive strain significantly reducing the oxygen exchange kinetics

Figure 1 shows the in-plane electronic transport properties for a series of fully strained LSCO films (20 nm in thickness) grown on LAO and LSAO followed by in situ post-annealing under vacuum and oxygen atmospheres. Each sample was annealed for different lengths of time to clarify the strain-driven kinetic behavior for each strain state. Because the loss of superconductivity is an indicator of increased oxygen nonstoichiometry, a fully oxygenated film will exhibit zero resistance below *T*_c_, whereas an oxygen-deficient film will show a decreased *T*_c_ or no superconductivity at all owing to hole depletion.

As shown in Fig. 1a, the as-grown (no post-annealing) LSCO film on LAO displayed insulating behavior. The observation of suppressed superconductivity in these cuprates was previously understood as a consequence of mechanical buckling owing to the tensile strain^18^. However, tensile-strained LSCO films post-annealed in oxygen (PO_2_ = 100 Torr) at 500 °C exhibited a gradual decrease in resistivity with increasing annealing time. Superconductivity was eventually turned on when they were annealed for 30 minutes or longer. This result ultimately indicates that there is an intrinsic oxygen deficiency when films are grown under tensile strain for a wide range of typical growth conditions. Consistent with this understanding, tensile-strained films after vacuum annealing at PO_2_ = 10^−6^ Torr for 30 min showed an even higher resistivity than the as-grown state, further confirming that the change in resistivity originated from variations in the oxygen stoichiometry, which are also known to influence the ground state of the lattice.

In the case of films under compressive strain, the result was markedly different, as shown in Fig. 1b. The as-grown LSCO film on LSAO was superconducting despite being grown under the same thermodynamic (temperature and oxygen pressure) conditions as the tensile-strained film grown on LAO. Thus, compressive-strained films showed an inherent stability of the superconductivity. To understand the origin of this stability, we post-annealed the compressive-strained films at 500 °C in 100 Torr of oxygen for 30 min. They showed very little change in resistivity. This observation suggests that compressively strained films are already fully oxygenated (or very nearly so) even in the as-grown state. LSCO on LSAO vacuum-annealed for 30 min at 500 °C showed very little change in the superconducting *T*_c_, unlike the tensile-strained analog, which showed an increase in resistivity when annealed in vacuum. Thus, under the same conditions, compressive-strained films show increased robustness against oxygen loss. To further investigate, a subset of compressive-strained LSCO films were vacuum-annealed for longer periods of time (0.5 to 6 h) to determine whether the removal of oxygen in the compressive-strained films is kinetically limited (i.e., whether a longer annealing time is required to create an oxygen deficiency). As shown in Fig. 1b, the loss of superconductivity occurred for compressive-strained films after a remarkably long post-annealing time of over 2 h—a significantly longer time than for LSCO on LAO, which took only 10 min to reach a comparable resistivity. Note that the overall film quality remained essentially unchanged even after six hours of post-annealing in vacuum, which strongly suggests that structural defects or phase impurities are not the main causes of suppressed superconductivity (but does not rule them out). Rather, this transport behavior implies that the oxygen exchange kinetics, which have been explored very little in these strained epitaxial films, play a critical role in determining the oxygen stoichiometry as well as the superconductivity. To summarize, we believe the main reason for the different oxygen instability between tensile-strained and compressive-strained films is due to the different oxygen surface exchange kinetics inherent in films, i.e., tensile strained (faster kinetics, more oxygen loss after vacuum annealing) versus compressively strained (slower kinetics, less oxygen loss after vacuum annealing for the same amount of time).

Even though the resistivity values of compressive-strained and tensile-strained films after post-annealing in oxygen were similar to each other, insight regarding the coupling of strain and superconductivity can be found by comparing the *T*_c_. The superconducting *T*_c_ of the oxygen post-annealed tensile-strained films (~7 K) was much lower than that of compressive-strained films (~31 K), implying that strain does play a role in controlling superconductivity. Moreover, this higher *T*_c_ of 31 K was much closer to the bulk value of 40 K, which can be realized only in a sample that is fully oxidized and without strain^1, 6^. That being said, the fact that very little change in *T*_c_ was observed for the oxidized and as-grown compressive-strained films, whereas the tensile-strained films showed a substantially decreased *T*_c_ for the as-grown films, emphasizes the inherent strain-induced oxygen loss for the latter.

### Electronic structure modification due to vacancy formation

**Fig. 2:**
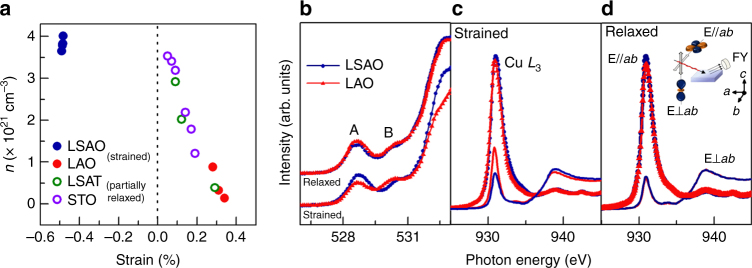
Strain-dependence and oxygen-dependence of the electronic structure. **a** Carrier density modulation determined at 50 K from Hall measurements from strained films grown on LSAO and LAO (closed circles) and partially relaxed films grown on LSAT and STO (open circles) to achieve different non-0% strain values. Gradual relaxation of tensile strain increases the hole carriers, approaching values that are similar to both strained and relaxed films under compressive strain. **b** Thickness-dependent XAS of the O *K* edge pre-peak region from strained (20 nm in thickness) and relaxed (125 nm) films (0% strain). The shift in spectral weight from peak A to peak B in strained films indicates hole depletion for tensile-strained films, which is not shown for the relaxed films on the same substrate. **c**, **d** Polarized XAS spectra of Cu *L*_3_ edge for the same set of films measured in **b**, where **E**⊥*ab* (lines) and **E**∥*ab* (symbols) are the electric field oriented with respect to the $3d_{z^2 - r^2}$ and $3d_{x^2 - y^2}$ orbitals as schematically shown in the inset

Although the electronic transport data provide compelling evidence for oxygen nonstoichiometry, we provide a thorough analysis of the strain-dependent carrier density as well as X-ray absorption spectroscopy (XAS) results, to further support our observations. From Hall measurements, we found that the electrical properties observed with different post-annealing conditions were associated with variations in the hole carrier concentration (Supplementary Fig. 2). A previous study has shown that strain relaxation may lead to films that are oxygen-stoichiometric (as shown by the higher *T*_c_)^19^. To expand upon this, we grew LSCO films (as-grown, no post-annealing) with different thicknesses on STO, LSAT, LAO, and LSAO to produce films with a broad range of strains. The charge carrier density, *n*, at 50 K from Hall measurements was calculated for each film (Fig. 2a). From these results, two key observations were made. The first of these observation is that strained films under compression and relaxed films under tensile strain had similar carrier densities. The second point is that increased tensile strain led to a substantially reduced carrier density. This trend of decreasing carrier density with increasing tensile strain indicates that the oxygen exchange kinetics for this material are significantly enhanced, directly impacting the number of oxygen vacancies present. As strain was released when films grew thicker, a more oxygen-stoichiometric film was produced owing to the slow oxygen exchange kinetics.

To relate our observations more directly to changes in electronic structure, we grew a set of strained (20 nm) and relaxed (125 nm) LSCO films on LSAO (compressive strain) and LAO (tensile strain) under “as-grown” conditions (no post-annealing) and probed their electronic structure using bulk-sensitive, polarization-dependent XAS. Figure 2b shows fluorescence yield (FY) XAS spectra of the O *K*-edge pre-peak region for both sets of films. For tensile-strained films, we observed a spectral weight transfer from peak A, the presence of which was attributed to doping-induced holes, to the upper Hubbard band (peak B) common to under-doped La_2−*x*_Sr_*x*_CuO_4_^20–22^. This spectral weight transfer suggests that as-grown, tensile-strained LSCO had a more hole-depleted state (i.e., more oxygen vacancies) than compressively strained LSCO. Additionally, Cu *L*-edge spectra are shown in Fig. 2c, d for the same set of films, where **E**⊥*ab* and **E**∥*ab* represent the polarization-dependent spectra collected along the out-of-plane and in-plane directions, respectively. The Cu *L*_3_ peak intensity for **E∥***ab* was similar for all films, indicating that the number of unoccupied $3d_{x^2 - y^2}$ states was nominally the same. Consistent with other Ruddlesden–Popper (2-1-4) cuprates, the overall weight of the unoccupied $3d_{x^2 - y^2}$ states was larger than that of the $3d_{z^2 - r^2}$ states^20^. Furthermore, relaxed and compressive-strained LSCO exhibit similar absorption spectra for both polarization configurations, which is remarkably consistent with the fairly similar hole carrier density (i.e., oxygen content and kinetic behavior) determined for these films. However, fully tensile-strained LSCO contains additional$3d_{z^2 - r^2}$ states not observed in the other three films. Since we have considerable evidence for a hole-depleted state in tensile-strained LSCO, the polarized XAS data strongly support that the density of states, specifically the distribution of holes, in these films is dictated by phenomena different from those that dominate in the chemically substituted cuprates. Although it is not the main focus of this work, this behavior could be related to a reduced Cu–O_apical_ distance induced by tensile strain, which is known to lower *T*_c_^23^. Since the films were grown under identical conditions, we reiterate that the observed changes in the absorption spectra under these as-grown conditions were driven by the different kinetic time scales for oxygen loss between tensile-strained and compressive-strained LSCO.

### Lattice response of strained LSCO films

**Fig. 3:**
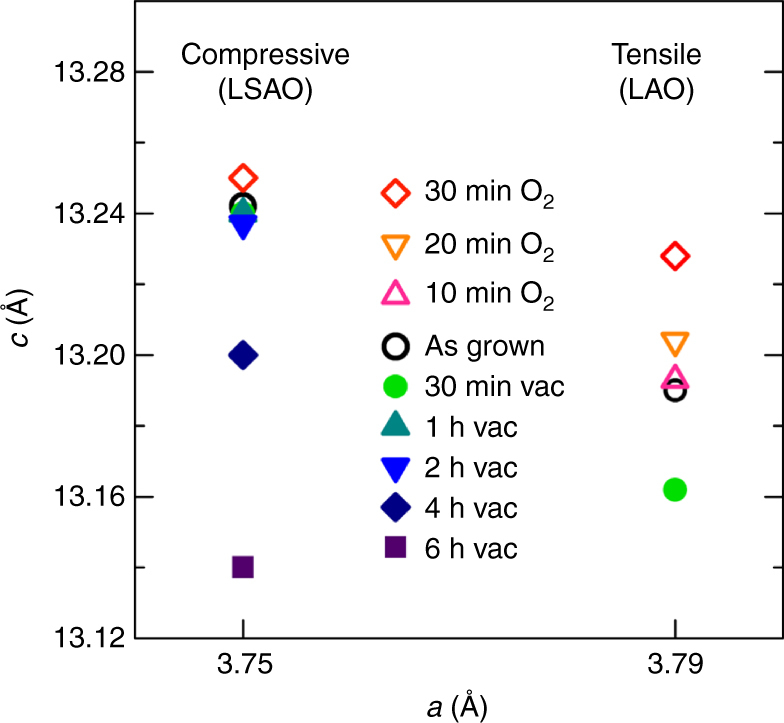
Redox sensitivity of lattice. Out-of-plane *c* lattice constants of 20-nm-thick LSCO films on LAO (tensile strain) and LSAO (compression) substrates. The lattice constants were measured from as-grown films and from films oxygen-annealed and vacuum-annealed for different time intervals at 500 °C. The in-plane lattice is fixed with the substrate in all films, as confirmed by X-ray reciprocal space mapping. Compressive-strained films show little sensitivity to oxygen annealing (closed circles) or vacuum annealing (triangles) for up to 2 h, after which a lattice reduction is observed. Tensile-strained films reveal that oxygen annealing leads to a gradual increase in the lattice parameter, whereas vacuum annealing for 30 min shows a significant lattice contraction, indicating the corresponding redox response of lattice volume changes

The previously discussed data describe the behavior of the electronic structure as a function of the strain state and the contrasting time required to form oxygen vacancies. Another important observation is the strain-dependent lattice constant change, which is critical for accurate determination of the types of oxygen defects. For perovskite oxides, the formation of oxygen vacancies results in lattice expansion^15, 24–28^. Thus, it was quite surprising that we observed the opposite lattice behavior, i.e., a 0.2% lattice contraction, upon post-annealing the Ruddlesden–Popper cuprate films in vacuum for 30 min. (Fig. 3). The compressive-strained films showed no obvious changes in *c* after annealing under the same conditions, consistent with the stable superconductivity and oxygen stoichiometry observed. However, post-annealing in vacuum for more than 2 h revealed a lattice contraction consistent with that in the oxygen-deficient films under tensile strain. This behavior strongly suggests that the compressive-strained films do not prohibit oxygen vacancy formation as is commonly suggested, but rather the surface exchange kinetics are much slower than in tensile-strained Ruddlesden–Popper phases. Moreover, the opposite trend, lattice expansion, was observed upon the filling of oxygen vacancies and/or creation of oxygen interstitials in tensile-strained films. In agreement with the previous discussion of the transport properties, no significant lattice expansion was observed when the film under compressive strain was annealed in oxygen. This result was due to a lack of change in the oxygen stoichiometry resulting from the very slow surface exchange kinetics. We found that a 30 min anneal was needed to realize a stable lattice constant, indicating that the film reached full oxidation after 30 min at 500 °C, as also was confirmed by the transport measurements.

### Energetics and kinetics of oxygen defects from calculations

It is evident that the variation in hole carrier concentration was a result of the different oxygen stoichiometries among compressive-strained and tensile-strained films. Such a difference in oxygen stoichiometry could be attributed to a change in the ability of LSCO to form oxygen defects during annealing under different redox conditions, or to a change in the oxygen surface exchange kinetics that govern the rate of oxygen incorporation into the film, or both. We reiterate that we found our films to have excellent structural integrity under all post-annealing conditions. To gain further insight into which mechanisms are dominant as a function of both strain and annealing conditions, we used DFT methods to calculate the oxygen chemical potential *µ*_O_ (Supplementary Table 1) and the oxygen-defect energetics. Known surface exchange data were also used to investigate the kinetics of oxygen incorporation in LSCO. Detailed calculation methods can be found in the Supplementary Note 2.

**Fig. 4:**
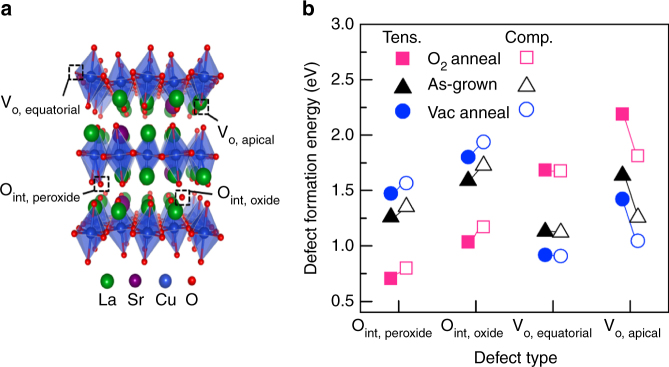
La_1.85_Sr_0.15_CuO_4_ defect positions and energetics. DFT-calculated defect formation energies for the four oxygen-defect types under experimental strain and annealing conditions. **a** the LSCO structure with the positions of the different oxygen defects labeled. **b** The plot of defect formation energy of each defect type for −0.61% compressive-strained and +0.29% tensile-strained films. Each pair of data points represents defect formation energies for interstitial peroxide (O_int, peroxide_), interstitial oxide (O_int, oxide_), equatorial oxygen vacancies (V_o, equatorial_), and apical oxygen vacancies (V_o, apical_) under different film annealing conditions: O_2_ annealing (500 °C, PO_2_ = 100 Torr), as-grown (700 °C, PO_2_ = 100 mTorr), and vacuum annealing (500 °C, PO_2_ = 10^−6^ Torr)

Figure 4a shows the positions of the different oxygen defects in LSCO considered (two vacancies: equatorial (V_O, equatorial_) and apical (V_O, apical_) and two interstitials: oxide (O_int, oxide_) and peroxide (O_int, peroxide_). A plot of these defect formation energies under different strain and annealing conditions is shown in Fig. 4b and summarized in Supplementary Table 2. From Fig. 4, we can extract several key points. First, the interstitial defects have lower formation energies under oxidizing conditions, whereas oxygen vacancy defects have lower formation energies under reducing conditions. Additionally, equatorial vacancies are more stable than apical vacancies under all conditions and peroxide interstitials (O_int, peroxide_) are more stable than oxide ion interstitials (O_int, oxide_). Finally, the relative differences in the formation energy of the stable interstitial (O_int, peroxide_) and stable vacancy (V_O, equatorial_) species between tensile and compressive strain is small, on the order of 100 meV. These findings are consistent with a previous DFT study examining oxygen defects in several strontium-doped Ruddlesden–Popper materials^29^. For the as-grown films, equatorial vacancies are expected to be the most abundant defect type for both tensile-strained and compressive-strained films. If we correlate these defect trends with the experimental data, a good understanding of the oxygen-defect chemistry can be achieved. For example, our data show that as the annealing conditions became more oxidizing, the measured *c*-axis for strained films increased, especially for tensile-strained films; the increase resulted from the filling of oxygen vacancies formed during the growth and/or the creation of oxygen interstitials. Calculations of the volume relaxation tensor^30^ under unstrained conditions for these defects (Supplementary Table 3) demonstrated that interstitials (both oxide and peroxide) resulted in *c* axis lattice expansion (*V*_cc_ > 0), whereas both apical and equatorial oxygen vacancies resulted in *c*-axis lattice contraction (*V*_cc_ < 0) (Supplementary Note 2). Therefore, in progressing from reducing to oxidizing conditions, we expect a loss of vacancies or gain of interstitials to result in lattice expansion. We note here that the effect of lattice contraction with the addition of vacancies is the result of redox of O atoms in LSCO, which is different from what happens in perovskite materials where redox occurs primarily on the transition metal sites. A more detailed explanation of these effects is provided in Supplementary Note 2 and Supplementary Table 4.

The measured *c*-axis changes in compressive-strained LSCO showed less variation under different annealing conditions than in tensile-strained LSCO. Because of the comparatively small variation under compression, it was evident that the kinetic limitations of oxygen incorporation were more pronounced in compression than in tension. Although the thermodynamics of defect formation could also play a role in the differences observed between the two strain states, the formation energy differences for the two favorable defects (i.e., peroxide interstitials and equatorial vacancies) in each case were very small, only on the order of 100 meV. Thus, a lower defect formation energy cannot be the dominant cause of the large differences in oxygen stoichiometry between compressive-strained and tensile-strained films observed in this work, although such changes may be dominant in perovskites^15, 16, 31^.

**Fig. 5:**
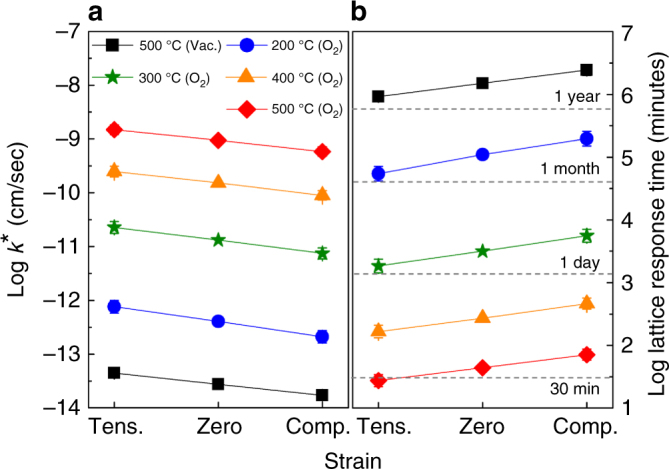
Strain-dependent oxygen kinetics. Variation of predicted oxygen surface exchange rate k* (**a**) and time required to incorporate oxygen (**b**) under different annealing conditions and strain states. Plot of the range of the approximated values of k* and time to fill the required number of oxygen sites in order to realize the measured *c*-axis lattice constants from Fig. 3 as a function of annealing condition and strain. Overall, k* is larger in tension than in compression. Conversely, the shortest oxygen filling times correspond to tensile strain, and the longest times to compressive strain as shown in **b**. The annealing conditions correspond to those used in experiments and the error bars were calculated based on approximate changes in the activation barrier for k* as a function of strain using previously published work on related materials (see Supplementary Note 3 for additional details)

Based upon the previous discussion, it is clear that defect formation energy was not the driving force behind the strain-driven phenomena observed in our films. Rather, we found that the oxygen surface exchange coefficient (k*) as a function of PO_2_, temperature, and strain provided the compelling evidence. Shown in Fig. 5a, b are approximate estimated values of k* (Supplementary Table 5) and the corresponding approximate lattice response times (Supplementary Table 6) for each annealing condition and strain state, respectively. Our usage of the term “lattice response time” represents the approximate time required for the lattice to fill or empty the required number of O sites in order to reach equilibrium after a certain temperatures and PO_2_ has been experimentally set. We note here that the estimated k* values in Fig. 5 are approximate, qualitative, and speculative in nature. This is because a series of approximations were required in order to estimate k* for different strain states as a function of temperature (see Supplementary Note 3 for more details). However, comparison of these approximately estimated k* values is still significant as these k* values produce qualitatively different timescales of lattice response time between tension and compression, which is fully consistent with the experimental results discussed above. A range of temperatures is provided in order to decouple the kinetic and thermodynamic effects. The range of approximate estimated k* values for the different strain states were obtained using the estimate of 89 meV/(% strain) variation in the activation barrier for k*. This estimate was based on the maximum difference in the calculated activation barriers for O migration with strain for an array of perovskite materials^32^(additional details on how k* was approximated can be found Supplementary Note 3). It is important to note that the as-grown state was used as the reference for the calculations of k*; thus, the computed lattice response times are all relative to the initial, equilibrium as-grown state. In support of our experimental data, tensile-strained LSCO had a larger estimated k* than did compressively strained LSCO under all conditions, suggesting that a shorter length of time was needed for oxygen incorporation under tension, as shown in Fig. 5b. The most reducing condition (500 °C in vacuum) had the lowest estimated k* value because of the low oxygen pressure, making it highly unfavorable for incorporating oxygen into the lattice. In fact, the calculated estimate of the lattice response time required to incorporate oxygen and achieve the lattice parameter plotted in Fig. 3 is over a year. It is not until the temperature is increased above 300 °C that the annealing times become experimentally feasible. Examination of these data emphasized the salient point that tensile strain resulted in a faster surface exchange rate by nearly an order of magnitude, and thus a smaller timescale, than compressive strain, regardless of temperature or pressure. Thus, control of oxygen stoichiometry in strained LSCO was governed primarily by the kinetics of oxygen surface exchange and not by the thermodynamics of defect formation. We believe that it is the change in activation energy of the surface exchange, presumably associated with an O_2_ splitting and an oxygen interstitial moving into the lattice, that gives rise to the strain dependence. This understanding of the oxygen kinetics is highly important in practical experiments and in preparing oxide materials with the desired oxygen stoichiometry.

## Discussion

Many critical physical properties and behaviors of complex oxides are not fully understood owing to the difficulty in accurately determining oxygen stoichiometry. Identifying the physical mechanism governing the value of the stoichiometry is an additional challenge. For example, it is generally believed that growing oxide thin films in high-oxygen partial pressure (on the order of 0.1 Torr) is sufficient to achieve full oxidization. However, as we report here, the strain state of the thin film plays a critical role for oxidation because the surface exchange kinetics for compressive and tensile strain can vary by an order of magnitude. Also, the kinetic behavior was found to be the dominant factor in determining the oxygen stoichiometry of these epitaxial thin films. This finding is in contrast to the current perception, which is that oxygen changes in epitaxial thin films such as these are generally the result of tensile strain causing a thermodynamically driven lower oxygen vacancy concentration (which does occur, but is a minor effect in our films). As many oxides exhibit drastic changes in their physical properties owing to strain, these results have significant implications not only for cuprate superconductors but also for many other multifunctional oxides. Moreover, the valuable understanding of how the kinetic behavior of oxygen loss affects the superconducting properties of LSCO suggests a new model for controlling *T*_c_; it goes well beyond assumptions that tensile strain favors oxygen vacancy formation, whereas compressive strain does not. The knowledge that we provide herein overturns the previous assumption that strain-induced structural deformation leads to the changes in *T*_c_^12–14^. Thus, deciphering the role of surface exchange kinetics coupled with strain is critical for identifying the true nature of physical property changes in a multitude of functional oxide thin films.

## Methods

### Experimental

We deposited optimally doped LSCO films by pulsed laser epitaxy. Note that the strontium content was nominally equivalent to the target, although the real film composition might have been slightly off owing to the highly energetic ablation process. Among various growth conditions, we systematically changed the lattice constants of the substrates to change both the sign of the strain (i.e., either tensile or compressive) and the degree by using various perovskite-based substrates, as stated in the main text. LSCO films were grown in an oxygen atmosphere of 0.1 Torr of O_2_ at a growth temperature of 700 °C. For the as-grown samples, films were quenched in 100 Torr O_2_ and cooled to room temperature. For the post-annealing protocols (vacuum and O_2_ annealing at PO_2_ = 10^−6^ and 100 Torr, respectively), samples were cooled to 500 °C and then post-annealed for 10 min, 20 min, 30 min, 1, 2, 4, or 6 h. The samples were cooled to room temperature in the atmosphere used for post-annealing. The laser fluence was fixed to 0.5 J/cm^2^. Structural data were obtained using a four-circle Panalytical X’pert Pro diffractometer. Representative XRD data, including *θ*−2*θ* scans and reciprocal space maps, of the high-quality LSCO films discussed in the text are illustrated in Supplementary Fig. 1. Transport measurements were obtained using a Quantum Design physical property measurement system using the typical Van der Pauw geometry. The electronic structure of strained LSCO films was probed using FY XAS performed at beam line 4-ID-C of the Advanced Photon Source at Argonne National Laboratory.

### Computational

All calculations were performed using DFT within the Vienna Ab Initio Simulation Package (VASP) with a plane-wave basis set^33^. We used the generalized gradient approximation exchange and correlation functional with Hubbard *U* correction (GGA + *U*)^34^ with the projector augmented wave method^35^ and pseudopotentials of Perdew, Burke, and Ernzerhof^36^. The Hubbard *U* correction was applied to copper atoms only, with *U* = 4 eV and *J* = 0 eV following Xie et al.^29^. The valence electron configurations for La, Sr, Cu, and O elements used were La: 5*s*^2^5*p*^6^6*s*^2^5*d*^1^, Sr: 4*s*^2^4*p*^6^5*s*^2^, Cu: 3*p*^6^3*d*^10^4*s*^1^, and O: 2*s*^2^2*p*^4^. All calculations were performed with spin polarization enabled. Reciprocal space integration in the Brillouin zone was conducted with the Monkhorst-Pack scheme^37^ with 4 × 4 × 2 k-point sampling. La_1.85_Sr_0.15_CuO_4_ cells were modeled as the low-temperature orthorhombic phase with space group *Bmab* (space group 64) with 112 atoms/cell. The positions of strontium atoms and defects in La_1.85_Sr_0.15_CuO_4_ were taken from Xie et al.^29^, who used the special quasirandom structure method to determine the placement of strontium dopants^38^. For the two types of O interstitial defects, the defects were inserted into the rock salt plane, and the oxide interstitial was positioned at approximate reduced cell coordinates of (0.15, 0.13, 0.25) (≈2.3 Å away from the nearest apical O) and the peroxide interstitial was positioned at approximate reduced cell coordinates of (0.18, 0.18, 0.21) (≈ 1.5 Å away from the nearest apical O), consistent with the work of Xie et al.^29^ Defect formation energy calculations were performed using standard defect thermodynamics models, as detailed elsewhere^29, 39^. In the cases where defects were calculated with lattice strain, the strain was imposed on the *a*-axis and *b*-axis lattice parameters, and the *c*-axis was allowed to fully relax. The volume relaxation tensors were calculated using the method of Centoni et al.^30^.

### Code availability

The VASP code, which is available for purchase, was used for the calculations in this paper. The configuration files used in these calculations are all available upon request from the authors.

### Data availability

The data that support the findings of this study are available from the corresponding author upon request.

## Electronic supplementary material

Supplementary Information
