# Supplementary material for: Strain control of oxygen kinetics in the Ruddlesden-Popper oxide La1.85Sr0.15CuO4
Source: Nat Commun. 2018 Jan 8;9:92. doi: 10.1038/s41467-017-02568-z (PMC5758782; doi:10.1038/s41467-017-02568-z)
Supplement: Supplementary file 1 — Supplementary Information [file 41467_2017_2568_MOESM1_ESM.pdf]

Supplementary Note 1

Experimental Details

We deposited optimally doped LSCO films by pulsed laser epitaxy. Note that the strontium content was nominally equivalent to the target, although the real film composition might have been slightly off owing to the highly energetic ablation process. Among various growth conditions, we systematically changed the lattice constants of the substrates to change both the sign (i.e., either tensile or compressive) and degree of the strain by using various perovskite-based substrates, as stated in the main text. For the growth of LSCO films, an oxygen atmosphere of 100 mTorr of O_2 at a growth temperature of 700 °C was optimized. For the as-grown samples, films were quenched in 100 Torr O_2 and cooled to room temperature. For the post-annealing protocols (10^{-6} Torr and 100 Torr O_2 annealing), samples were cooled to the annealing temperature (100, 200, 300, 400, or 500 °C) and then post-annealed for 10 min, 20 min, 30 min, 1 h, 2 h, 4 h, or 6 h. The samples were cooled to room temperature in the atmosphere used for post-annealing. The laser energy was fixed to 0.5 J/cm^2. Representative XRD spectra and reciprocal space mapping data, obtained from a four-circle Panalytical X'pert Pro diffractometer, of the high-quality LSCO films discussed in the text were collected for each film and are illustrated in Supplementary Fig. 1. The *c*-lattice parameters were determined from an average of the 006 and 008 d-spacing values. The in-plane (*a*) lattice parameters were determined by identifying the reciprocal space vectors, q_x and q_z, central position for both the substrate and the film epitaxially-related *hkl* reflections. For a fully strained film, q_x of the substrate equal q_x of the film (example shown in Supplementary Fig. 1). The values were then used to determine the in-plane lattice parameter according to the following equation:

$$a_{film} = \left[\frac{\left(\frac{q_{x\,(sub)}}{h_{sub}}\right)}{\left(\frac{q_{x\,(film)}}{h_{film}}\right)}\right] x\ a_{sub} \qquad (1)$$

The in-plane strain was then determined by:

$$\text{in} - \text{plane strain} = \frac{a_{sub} - a_{film}}{a_{sub}} \qquad (2)$$

Transport measurements were obtained using a Quantum Design physical property measurement system using the typical Van der Pauw geometry. Temperature-dependent Hall coefficient of the annealed films are illustrated in Supplementary Fig. 2. The electronic structure of strained LSCO films was probed using fluorescence yield X-ray absorption spectroscopy performed at beam line 4-ID-C of the Advanced Photon Source at Argonne National Laboratory.

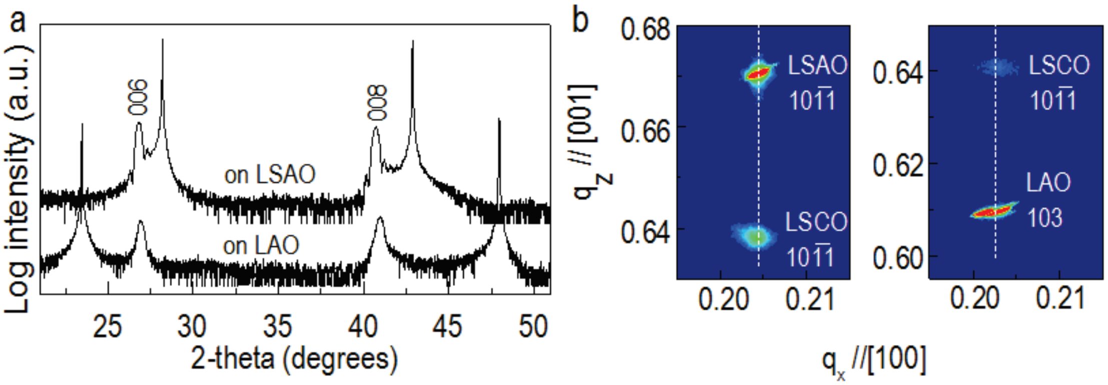

Supplementary Figure 1. Structural characteristics of epitaxial $La_{1.85}Sr_{0.15}CuO_4$ films. a, X-ray diffraction spectra of LSCO films grown on LSAO (a = 3.756 Å) and LAO (a = 3.788 Å) single-crystal substrates. The (00*l*) peaks of LSCO film on each substrate show that the LSCO is grown along the (001) direction and confirms the high quality of the films and absence of secondary phases. **b**, Reciprocal space mapping of the (1 0 11) peak of LSCO films grown on each substrate illustrates the fully strained state of the LSCO.

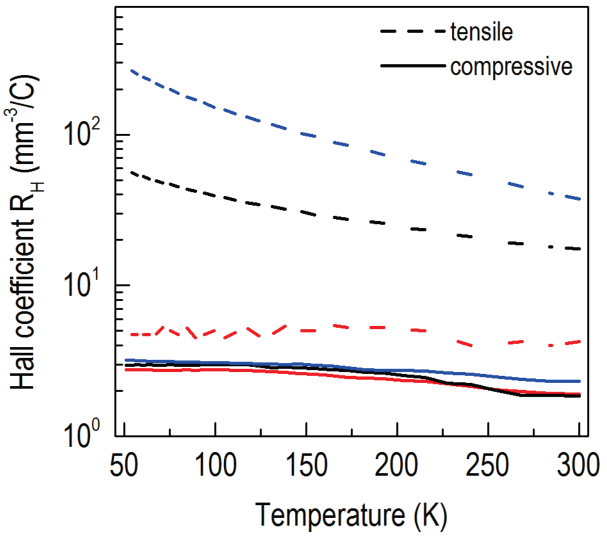

Supplementary Figure 2. Strain and oxygen-dependent Hall coefficient. Hall coefficient strained LSCO films grown on LAO (tensile) and LSAO (compressive). Compressively strained films show little change in the Hall coefficient, whereas tensile-strained films show a strong deviation among oxygen-annealed (red), as-grown (black), and vacuum-annealed (blue).

Supplementary Note 2

Defect calculations: energetics and formation volumes

Our approach to calculating defect formation energies in the present work uses the well-established defect thermodynamics methods used in previous works.[1-3] Briefly, the defect formation energy ΔE^{α}_{form} for oxygen defects of type α (vacancy or interstitial) in LSCO is defined as:

$$\Delta E^{\alpha}_{form} = E_{defected} - E_{perfect} \pm \mu_O \quad (3)$$

where $E_{defected}$ is the DFT energy of the defected LSCO cell, $E_{perfect}$ is the energy of the undefected LSCO cell, and μ_O is the calculated chemical potential of oxygen. The (+) sign is used when forming a vacancy and (–) sign is used for interstitials. The value for μ_O is temperature- and pressure-dependent and is calculated using the expression:

$$\mu_O(T,P) = \frac{1}{2}\left[E_{O_2}^{VASP} + \Delta h_{O_2} + H(T,P^0) - H(T^0,P^0) - TS(T,P^0) + kT\ln\left(\frac{P}{P^0}\right)\right] \quad (4)$$

where $E_{O_2}^{VASP}$ is the DFT calculated energy of an isolated O_2 molecule and Δh_{O_2} (Δh_{O_2} = 0.7023 eV/O, the value tabulated in the Materials Project) is the empirical oxygen energy shift to correct for the DFT overbinding of the O_2 molecule and temperature correction between 0 K and 298 K. The values $H(T,P^0)$, $H(T^0,P^0)$ and $S(T,P^0)$ are the gas enthalpy and entropy values at standard temperature T^0 = 298 K and pressure P^0 = 1 atm and arbitrary temperature *T*, and the logarithmic term in Eq. (4) is the change in μ_O as a function of pressure assuming an ideal gas. The gas enthalpy and entropy values were obtained using data from the National Institute for Standards and Technology (NIST) chemistry webbook.[4] The values of μ_O used in the defect calculations for each annealing condition are summarized below in Supplementary Table 1.

**Condition**	Vacuum anneal	As-grown	O_2 anneal
T **(°C)**	500	700	500
P **(Torr)**	10^{-6}	0.1	100
μ_O **(eV/O)**	-5.78	-5.72	-5.16

Supplementary Table 1. Calculated values of μ_O for different annealing conditions.

Using the computed values of μ_O from Supplementary Table 1, the defect formation energies of the four defect types discussed in the main text and shown in Fig. 4 are summarized in Supplementary Table 2.

**Defect type**	Oxide interstitial, $O_{int\ oxide}$		
**Condition**	O_2 anneal	As-grown	Vacuum anneal
**-0.61% compression**	1.52	2.07	2.13
**+0.29% tension**	1.18	1.73	1.79
**Defect type**	Peroxide interstitial, $O_{int,\ peroxide}$		
**Condition**	O_2 anneal	As-grown	Vacuum anneal
**-0.61% compression**	0.80	1.35	1.41
**+0.29% tension**	0.83	1.38	1.44
**Defect type**	Equatorial vacancy, $V_{O,\ equatorial}$		
**Condition**	O_2 anneal	As-grown	Vacuum anneal
**-0.61% compression**	1.68	1.12	1.06
**+0.29% tension**	1.71	1.15	1.09
**Defect type**	Apical vacancy, $V_{O,\ apical}$		
**Condition**	O_2 anneal	As-grown	Vacuum anneal
**-0.61% compression**	1.81	1.26	1.20
**+0.29% tension**	2.05	1.50	1.44

Supplementary Table 2. Calculated values of the defect formation energy for oxygen vacancies and interstitials under the three *(T, P)* conditions from Supplementary Table 1 and two strain conditions. The units of defect formation energies are all eV/defect.

To assess how the *c*-axis of LSCO is expected to change as the concentration of oxygen vacancies and interstitials changes under different annealing conditions, we calculated the

relaxation volume tensors for each of the four defect types under unstrained conditions. Following the method of Centoni *et al.*, the volume relaxation tensor V_{relax} can be calculated using the Expression[5]

$$V_{relax} = \det(L_0)\ln(L_0^{-1}L) \ , \tag{5}$$

where L_0 is the matrix of lattice constants for the relaxed, undefected cell, and L is the matrix of lattice constants of the relaxed cell containing the defect under study. Supplementary Table 3 summarizes the calculated values of the V_{CC} (*c*-axis) component of the volume relaxation tensor for each defect type. As stated in the main text, vacancies result in a *c*-axis reduction (especially for equatorial vacancies), and interstitials result in a *c*-axis expansion (especially for oxide interstitials).

**Defect Type**	**V_{cc} (Å^3)**
Oxide interstitial, $O_{int,\ oxide}$	3.0528
Peroxide interstitial, $O_{int,\ peroxide}$	1.9456
Equatorial vacancy, $V_{O,\ equatorial}$	-3.1920
Apical vacancy, $V_{O,\ apical}$	-2.2229

Supplementary Table 3. Calculated values of V_{CC} (*c*-axis components) of the volume relaxation tensor for each defect type under unstrained conditions.

From Supplementary Table 3, the formation volume of interstitials is positive, while the formation volume of vacancies is negative. The behavior of vacancies is different from what happens in perovskites, where forming vacancies results in an expansion of the lattice. Here, we have provided a brief discussion of the redox behavior of Ruddlesden-Popper La_2CuO_4 in relation to the more widely-studied perovskite materials. In, for example, perovskite $LaCuO_3$, Cu is in the 3+ oxidation state. When an O vacancy is created, it donates electrons to the lattice, which reduces Cu from 3+ to 2+, due to the instability of having Cu^{3+}. Generally, O vacancies in perovskites have

a positive formation volume. This is because the increase in size of the reduced transition metal dominates over the reduction in size by removing O.

In Ruddlesden-Popper La_2CuO_4, Cu is now in the 2+ oxidation state. When an O vacancy is created, the donated electrons can either go toward reducing Cu to the 1+ state, or to reducing O. Based on our Bader charge analysis, which is summarized in Supplementary Table 4, O vacancies primarily result in the reduction of O, not in reduction of Cu from 2+ to 1+, which is a result of the instability of Cu^{1+}. This is different from what happens in the perovskite, and we believe this is the origin of the negative formation volume for vacancies in the Ruddlesden-Popper phase.

Defect type	Charge difference (defected – perfect) (e^-/cell)	Fraction of charge difference from redox of O
Oxide interstitial, $O_{int, oxide}$	1.06	0.86
Peroxide interstitial, $O_{int, peroxide}$	0.72	0.95
Equatorial vacancy, $V_{O, equatorial}$	-1.34	0.63
Apical vacancy, $V_{O, apical}$	-1.34	0.62

Supplementary Table 4. Summary of Bader charge analysis for each defect type in the unstrained condition.

Supplementary Figure 3 contains plots of the calculated defect formation energy under as-grown conditions as a function of strain for each defect type. In general, the functional relationship between the defect formation energy with strain is quadratic. Overall, the variation in formation energy with strain is small, around 100-200 meV. In particular, for the stable vacancy and interstitial species, the variation of formation energy with strain is < 100 meV. We note here that at larger strain states, the variation in defect formation energy may become larger to the point where the defect energetics play a more sizable role in the measured stoichiometry of strained LSCO thin films. However, for the lower strain states considered in this study, the strain response of the

oxygen stoichiometry is due to changes in the oxygen surface exchange kinetics, and not due to the changes in the oxygen defect thermodynamics.

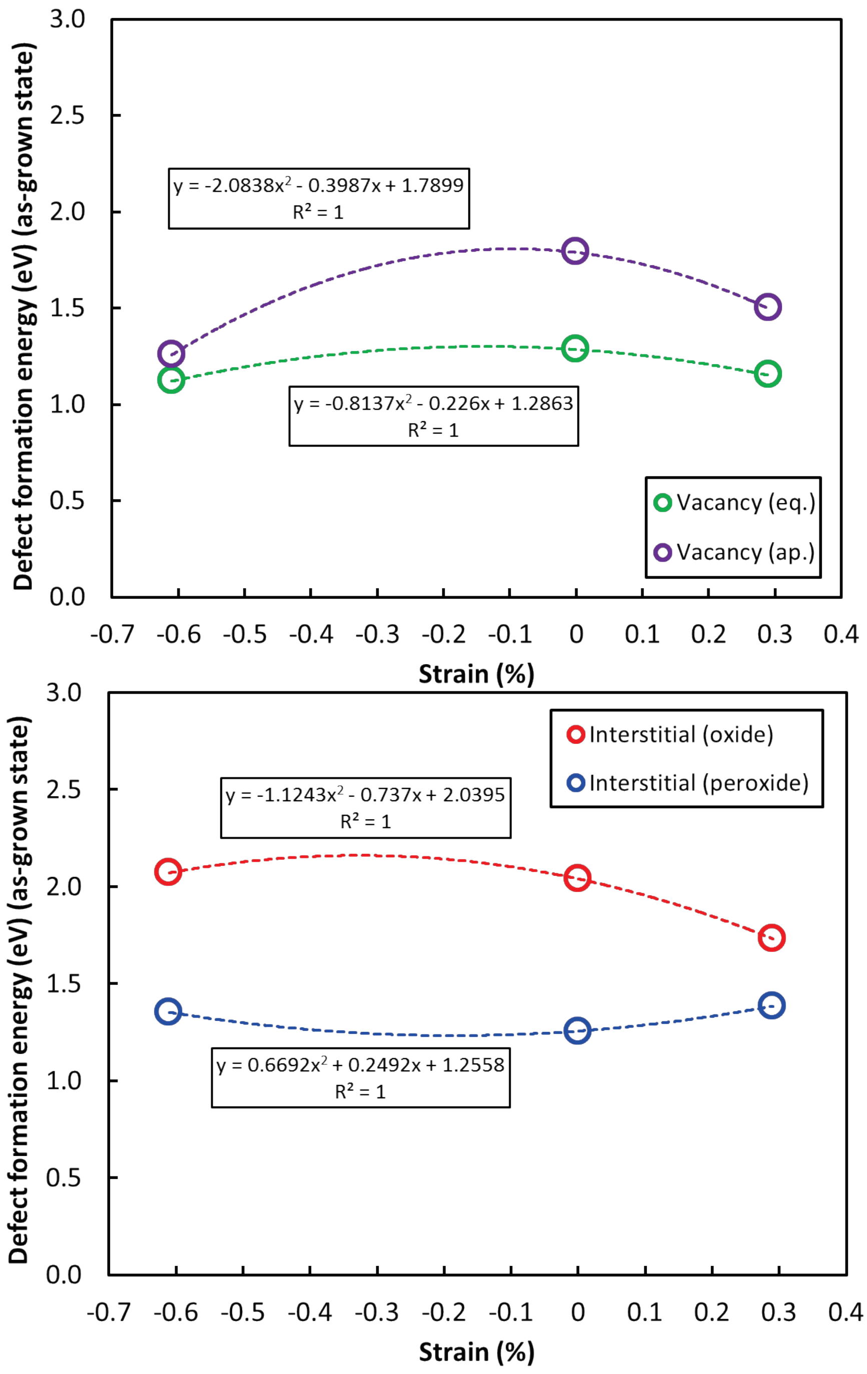

Supplementary Figure 3. Plots of defect formation energy under the as-grown state as a function of strain for vacancies (top panel) and interstitials (bottom panel).

Supplementary Note 3

Estimation of *k for LSCO under arbitrary (*T*, *P*), effect of strain and time to fill oxygen sites**

In this work, we drew upon previously published data for surface exchange in LSCO to compute qualitative, approximate *k** values as a function of temperature, pressure, and strain state. We used these computed values of *k** under each annealing condition to then estimate the time required to fill enough oxygen sites to result in the measured *c*-axis values reported in Fig. 3 of the main text. From the work of Claus et al.,[6] we extracted data of *k** as a function of temperature at ambient oxygen pressure $P(O_2) = 0.2$ atm. The Arrhenius relationship from the data in their work is:

$$k^* = \left(8.2235\frac{cm}{s}\right)\exp\left(\frac{-1.64eV}{k_B T}\right). \tag{6}$$

From the work of Lee, et al.,[7] the authors report the pressure dependence of *k** in LSCO as:

$$k_2^* = k_1^*\left(\frac{P_{O_2,2}}{P_{O_2,1}}\right)^{0.89}. \tag{7}$$

The samples in the current work and the work of Lee et al.[7] are thin films of LSCO. Therefore, we can use the measured *k** value from Lee, et al., Eq. (6) and Eq. (7) to obtain approximate, qualitative values of *k** at different *(T, P)*. As Eq. (6) was obtained from *k** measurements on polycrystalline LSCO, we are making the assumption that the activation barrier for *k** for polycrystalline and thin film samples is the same. This assumption is necessary because, to our knowledge, the activation barrier for thin film LSCO has not been experimentally measured, and is reasonable as our goal is only to show the approximate, qualitative differences in lattice response times between different annealing conditions and the qualitative effect of strain on *k** based on other reported materials data. As a sensitivity check, we find that the lattice response times vary by

about +/- 0.8 log units when the activation barrier for k^* varies by +/- 250 meV. This variation would not impact any of the qualitative conclusions of the study.

Supplementary Table 5 contains a summary of the calculated k^* values for each annealing condition. For the purposes of calculating the approximate time needed to fill the LSCO oxygen sites to obtain the *c*-axis values in Fig. 3 of the main text, we used the geometric averages of the k^* values under each annealing condition and the as-grown condition. The logic behind this choice was that since each film was grown under the as-grown conditions, it would have a value of k^* which, before further annealing, began with a k^* value from the as-grown conditions. Then, as the (T, P) were changed to anneal at any other condition, k^* would change over time to become the k^* value computed for that final annealing condition. As k^* may change by orders of magnitude between annealing conditions, and as it is not known how quickly k^* evolves when subjected to a new (T, P), the use of the geometric average is a reasonable approach to include contributions from k^* of the as-grown state and the chosen ending state. The values of k^* listed in Supplementary Table 5 that correspond to the geometric average with the as-grown state are the data points for zero strain plotted in Fig. 5 of the main text.

***T* (°C)**	500	700 (as-grown state)	500	400	300	200
***P* (Torr)**	10^{-8}	0.1	100	100	100	100
***k** (cm/s)**	1.71×10^{-18}	4.46×10^{-10}	1.97×10^{-9}	5.18×10^{-11}	3.85×10^{-13}	3.63×10^{-16}
***k** (geometric average with as-grown state) (cm/s)**	2.76×10^{-14}	4.46×10^{-10}	9.36×10^{-10}	1.52×10^{-10}	1.31×10^{-11}	4.02×10^{-13}

Supplementary Table 5. Computed values of *k for unstrained LSCO based on the extracted relationships and data from previously published work.** The data for k^* (geometric average with the as-grown state) are the data points for zero strain plotted in Figure 5 of the main text.

The last component for estimating the range of k^* is the effect of strain. To our knowledge, no

experiments have been performed that specifically measure the variation in *k** as a function of strain in any Ruddlesden-Popper material. Thus, the best approximation we can make is to draw upon data for a similar material, the perovskite $La_{1-x}Sr_xCoO_3$, and data of calculated activation barriers for O migration in perovskites to estimate a sensible bound on the variation in *k** as a function of strain. The work of Kubicek *et. al.*[8] on perovskite $La_{1-x}Sr_xCoO_3$ demonstrated that strain resulted in a *k** change of a factor of 4 between +1.4% tension and −1.5% compression, corresponding to a change in the activation barrier for surface exchange of about 40 meV, or about 13 meV/(% strain). For the upper bound of the activation barrier variation with strain, we use the value of 89 meV/(% strain), which was found to be the maximum difference in the calculated activation barriers for O migration for an array of perovskite materials as a function of strain.[9] We believe that, in the absence of strain-dependent *k** activation barrier data for Ruddlesden-Popper materials, this 89 meV/(% strain) bound on the activation barrier for *k** is a reasonable approximation because both surface exchange and bulk O migration are activated processes that involve moving oxygen in the lattice, and perovskites and Ruddlesden-Popper oxides are similar classes of materials. Furthermore, the oxygen activation volume (which governs the strain response) in perovskite is likely to be larger than the activation volume an interstitial incorporating in LSCO, and we have taken the largest strain response value over the wide-range of perovskites studied by Mayeshiba, et al.[9] Thus, the strain dependence of the activation barrier for *k** is bounded between approximately 13 and 89 meV/(% strain), where tension (compression) results in a higher (lower) value of *k**.

Although the values reported in Fig. 5 of the main text are very approximate, and quantitative only to within plus or minus one log unit, this range in values is on a par with typical experimental error bars among different sample preparations, measurement techniques, and equipment calibrations. Therefore, useful trends and qualitative behavior can be inferred from these

data over the range of different annealing and strain conditions considered herein. Now that the values of k^* under each annealing condition are known, the time required to fill the LSCO oxygen sites to produce the respective measured c-axis changes for each annealing condition can be calculated. To do this, we use a simple oxygen flux model, which results from Fick's first law following the work of van der Haar *et al.*: [10]

$$R_O = k^*\left(\left[O_{\text{int}}\right]-\left[O_{\text{int,equil.}}\right]\right) \tag{8}$$

where R_O is the flux of O passing through the surface in units of (#O/cm^2-s), $\left[O_{\text{int}}\right]$ is the concentration of oxygen interstitials at some *(T, P)* and $\left[O_{\text{int,equil.}}\right]$ is the equilibrium concentration of oxygen interstitials at some *(T, P)*, both in units of (#O/cm^3). From Fig. 3 of the main text, we examined the c-axis values under tension for each annealing environment, and looked at the c-axis change when progressing from the as-grown state to each other annealing state. The change in c-axis was directly related to the amount of oxygen added or removed from the lattice. Herein, we write the concentration difference in terms of number of interstitials per cubic centimeter. The approximate c-axis "height" of a peroxide interstitial (the most stable interstitial defect) was obtained from the volume relaxation tensor value in Supplementary Table 3 and assuming a spherical radius. Thus, the V_{cc} value of 1.95 Å^3 produces an interstitial radius of 0.77 Å. This value allows the conversion of a c-axis lattice change (in units of Å) to units of (#O/cell), which can easily be converted into units of (#O/cm^3) using the unit cell volume. The c-axis values from Fig. 3 of main text, the change in c-axis under different annealing conditions, and the corresponding change in oxygen interstitial concentration is summarized in Supplementary Table 6.

(T, P) (°C, Torr)	c-axis (Å)	c-axis change per cell from (T, P) = (700, 0.1) (Å)	$[O_i]$-$[O_{i,eq}]$, (# O ints/cm^3)	Time to fill all oxygen interstitial sites (k^* tension, k^* compression) (log min)
500, 100	13.199	0.040	3.36×10^{19}	1.34, 1.94
400, 100	13.188	0.029	2.44×10^{19}	2.12, 2.75
300, 100	13.173	0.014	1.18×10^{19}	3.15, 3.85
200, 100	13.168	0.009	7.56×10^{18}	4.62, 5.41
700, 0.1	13.159	0	0	n/a
500, 10^{-8}	13.130	-0.029	-2.44×10^{19}	5.89, 6.47

Supplementary Table 6. Values of measured LSCO *c*-axis from Figure 3 of the main text (plus additional values from annealing at other temperatures), the change in *c*-axis between the as-grown state and the other annealing conditions, the calculated change in number of oxygen interstitials required to realize the requisite change in *c*-axis, and the time required to fill all oxygen interstitials (in log minutes) assuming that LSCO has a range of k^* values corresponding to k^* in tension (higher k^*, shorter time to fill oxygen interstitials) and k^* in compression (lower k^*, longer time to fill oxygen interstitials).

Finally, now that the values of k^* and O interstitial concentration are in the correct units, the value of oxygen flux can be calculated and turned into units of time. The oxygen flux R_O has units of (#O/cm^2-s). Multiplying this value by the cross-sectional area of the films, which is $1.25\text{x}10^{-6}$ cm^2, we obtain an oxygen current in (#O/s). Inverting this quantity to (s/#O) and multiplying by the number of required interstitials and film volume (the films are 25 nm thick, so the volume is $3.125\text{x}10^{-12}$ cm^3) and converting to minutes yields the time needed to fill the necessary number of O sites in the lattice.

Supplementary References

1. Xie, W., Lee, Y.-L., Shao-Horn, Y. & Morgan, D. *The Journal of Physical Chemistry Letters* **7,** 1939–1944 (2016).
2. Jacobs, R. M., Booske, J. H. & Morgan D. *Physical Review B* **86,** 054106 (2012).
3. Lee, Y.-L., Kleis, J., Rossmeisl, J. & Morgan, D. *Physical Review B* **80,** 224101 (2009).
4. *NIST Standard Reference Database No. 69,* edited by Linstrom P. J. & Mallard W. G. (National Institute of Standards and Technology, Gaithersburg, MD, 2003). Available

from: http://webbook.nist.gov/chemistry/.
5. Centoni, S. A., Sadigh, B., Gilmer, G. H., Lenosky, T. J., Diaz de la Rubia, T. & Musgrave, C. B. *Physical Review B* **72,** 195206 (2005).
6. Claus, J., Borchardt, G., Weber, S., Hiver, J.-M. & Scherrer, S. *Materials Science and Engineering: B* **38,** 251–257 (1996).
7. Lee, Y.-L., Lee, D., Wang, X. R., Lee, H. N., Morgan, D. & Shao-Horn Y. *The Journal of Physical Chemistry Letters* **7,** 244–249 (2016).
8. Kubicek, M., Cai, Z., Ma, W., Yildiz, B., Hutter, H. & Fleig, J. *ACS Nano* **7,** 3276–3286 (2013).
9. Mayeshiba, T. & Morgan, D. *Physical Chemistry Chemical Physics* **17**, 2715-2721 (2015)
10. van der Haar, L. M., den Otter, M. W., Morskate, M., Bouwmeester H. J. M. & Verweij, H. *Journal of The Electrochemical Society* **149,** J41–J46 (2002).
